# The impact of *CYP2C9*, *VKORC1*, and *CYP4F2* polymorphisms on warfarin dose requirement in Saudi patients

**DOI:** 10.3389/fphar.2025.1547142

**Published:** 2025-04-30

**Authors:** Salha Jokhab, Maha M. AlRasheed, Dana Bakheet, Abdulkareem AlMomen, Nouf AlAboud, Farhad Kamali

**Affiliations:** ^1^ Department of Clinical Pharmacy, College of Pharmacy, King Saud University, Riyadh, Saudi Arabia; ^2^ DNA Extraction and Oligo Synthesis Unit, King Faisal Specialist Hospital and Research Center, Riyadh, Saudi Arabia; ^3^ Medicine-Hematology, King Khalid University Hospital, King Saud University, Riyadh, Saudi Arabia; ^4^ Translational and Clinical Research Institute, Newcastle University, Newcastle upon Tyne, United Kingdom

**Keywords:** warfarin, CYP2C9, VKORC1, CYP4F2, polymorphisms

## Abstract

**Background:**

Limited data are available on factors that affect warfarin dose requirement in Saudi patients. Saudis are among the underrepresented ethnic groups in warfarin pharmacogenetics research. The present study investigated the frequency of CYP2C9*2 and*3, *CYP4F2* (G1347A) and *VKORC1* –1639G>A genotypes and their impact on warfarin dose requirement in a cohort of Saudi patients requiring anticoagulation therapy.

**Methods:**

193 patients on chronic warfarin therapy and with stable anticoagulation took part in the study. Genotyping for *VKORC1* 1639G>A, *CYP4F2* G1347A, CYP2C9*2 430C>T and CYP2C9*3 1075A>C were performed using TaqMan genotyping assays. Analysis of variance was carried out to determine the association between *CYP2C9*, *CYP4F2,* and *VKORC1* genotype and warfarin dose requirement in two groups based on target INR range. Backward linear regression analysis identified genetic and clinical factors influencing doe requirements.

**Results:**

Patients with *CYP2C9* and *VKORC1* polymorphisms required significantly lower warfarin doses compared to wild-type patients. Carriers of two mutant alleles required lower doses than those with one mutant allele. In contrast, *CYP4F2* polymorphisms did not influence warfarin dose. Age and genetic variants in *CYP2C9* and *VKORC1* were negatively correlated with dose requirements, while body surface area (BSA) was positively correlated.

**Conclusion:**

Saudi patients with polymorphisms in *CYP2C9* and *VKORC1* required lower warfarin doses than those with the wild-type allele. *CYP4F2* polymorphism had no effect on warfarin dose requirement. Integrating patient clinical factors, including age and BSA, and genetic polymorphisms in *CYP2C9* and *VKORC1* provides the best estimation of factors contributing to warfarin dose in the Saudi patient population.

## 1 Introduction

For many years, vitamin K antagonists (VKAs), such as warfarin, have proven effective in preventing and treating arterial and venous thrombotic events. Warfarin exerts its anticoagulant effect via the inhibition of the vitamin K epoxide reductase (VKOR) enzyme, thus preventing reduction of vitamin K and reducing the synthesis of functional vitamin K–dependent clotting factors. Polymorphism in the *VKORC1* gene is a major contributor to warfarin dose requirement. This gene is located on chromosome 16 and encodes the VKOR enzyme. *VKORC1*-1639A variant allele is associated with decreased warfarin dose requirement and increased risk of over anticoagulation (Bourgeois et al., 2016; Sconce et al., 2005; Scott et al., 2010).

Warfarin is a 1:1 racemic mixture of the R and S stereoisomers. The S enantiomer is approximately five times more potent than the R warfarin (Moyer et al., 2009). CYP2C9 is the main enzyme involved in the metabolism of (S)-warfarin (Kalman et al., 2016; Westervelt et al., 2014). More than 57 allelic variants of CYP2C9 have been identified (Niinuma et al., 2014). CYP2C9*2 and CYP2C9*3 are the most common SNPs found to affect warfarin metabolism. Individuals carrying the CYP2C9*2 or CYP2C9*3 alleles usually have decreased enzymatic activity and therefore slower warfarin metabolism, decreased warfarin clearance, increase warfarin sensitivity and bleeding risk (Gulseth et al., 2009; Kamali and Wynne, 2010; Lane et al., 2012; Moyer et al., 2009; Sconce et al., 2005).

Monitoring therapeutic response to VKAs is necessary because of the risk of thrombosis and bleeding associated with under- and over-anticoagulation, respectively (Wigle et al., 2013). The International Normalized Ratio (INR) is the most common method for monitoring VKAs therapy. Deviations from target INR range increases the risk of bleeding or stroke particularly during the first weeks of therapy (Homme et al., 2008).

Achieving and maintaining optimal anticoagulation is challenging due to the wide inter- and intra-individual variation in warfarin response and dose requirements. Although over 60 years have passed since warfarin was first used as an anticoagulant, the factors that contribute to the variability in dose requirement (up to 20-fold in daily dose requirement) have not yet been fully identified (Wen and Lee, 2013). Demographic, clinical, and genetic factors are shown to contribute to warfarin dose requirement (Kamali and Wynne, 2010). *CYP2C9* and *VKORC1* are reported to be the most important genetic determinants of warfarin dose requirements in different patient populations (Borgiani et al., 2009; Cen et al., 2010; Tatarunas et al., 2014). Other genetic variations, including that in the *CYP4F2* gene, have been associated with warfarin dose but to a lesser extent. Genetic polymorphisms in *VKORC1 -1639G>A*, CYP2C9*2, and CYP2C9*3, together with clinical factors such as age and sex, explain up to 57% of the variability in warfarin dose requirement (Cavallari and Perera, 2012; McDonald et al., 2009; Zhang et al., 2017).

Significant differences exist in warfarin dose requirements between different ethnic groups, whereby Asians and African Americans require lower and higher warfarin doses, respectively, compared with Caucasians (Cavallari and Perera, 2012; Kamali and Wynne, 2010). Saudis are among the underrepresented ethnic groups in warfarin pharmacogenetics research. Only a few studies in Saudi patients have thus far reported the prevalence of *CYP2C9* and *VKORC1* polymorphisms and their impact on warfarin dose requirement and patient response (Al Ammari et al., 2020; Alzahrani et al., 2013; Mirghani et al., 2011; Mizzi et al., 2016; Saour et al., 2011; Al-Saikhan et al., 2018). To date, no studies have explored the effect of *CYP4F2* genetic polymorphisms on warfarin dose requirements in Saudi patients. The present study was designed to a) investigate the frequency of CYP2C9*2 and *3, *CYP4F2* (*G1347A*) and *VKORC1 –1639G>A* genotypes in the Saudi population, b) to evaluate the impact of polymorphisms in *CYP2C9*, *CYP4F2* and *VKORC1* genes on warfarin dose requirement, and c) to explore the association between CYP2C9*2,*3, *CYP4F2* (G1347A) and *VKORC1* –1639G>A genotypes along with other non-genetic factors and warfarin dose.

## 2 Materials and methods

### 2.1 Study design

This cross-sectional study was conducted at the King Khaled University Hospital (KKUH) anticoagulation clinic. Ethical approval was obtained from the Institutional Review Board in November 2017. All patients signed informed consent before participation. DNA extraction and genotyping were performed according to established methods at King Faisal Specialist Hospital and Research Centre (KFSHRC).

### 2.2 Participants

Between March 2018 and October 2019, 193 patients on warfarin therapy were recruited from the anticoagulation clinic at KKUH. The patients included were 18 years old or older, on at least 12 months of warfarin therapy, and had stable anticoagulation. A warfarin dose requirement that has been constant for at least three prior clinic visits spanning a minimum of 3 months and an INR within the target range are considered signs of stable anticoagulation. Patients were excluded if they had abnormal hepatic or renal function or were receiving medications known to interact with warfarin.

### 2.3 Data collection

Patient data were obtained using a self-report questionnaire and hospital records. The questionnaire comprised demographic information and warfarin-related information (indication, duration of use, recommended INR range). Demographic information comprised gender, age, weight, height, marital status, education, and place of residence. Data were also obtained from participants’ medical records, including comorbidities, concomitant medical therapy, previous stroke, and bleeding events.

### 2.4 DNA extraction and genotyping

DNA extraction was performed at KFSHRC according to the manufacturer’s protocol using the PureGene DNA extraction kit (Qiagen Sciences, Germantown, Maryland, United States). A Nanodrop ND-1000 spectrophotometer (Wilmington, DE, United States) was used to determine DNA concentration and purity. Genotyping for *VKORC1* 1639G>A (rs9923231; C_30403261_20), *CYP4F2* G1347A (rs2108622; C_16179493_40), CYP2C9*2 430C>T (rs1799853; C_25625805_10) and CYP2C9*3 1075A>C (rs1057910; C_27104892_10) was performed using TaqMan genotyping assays according to the standard protocol provided by Applied Biosystems. The TaqMan real-time polymerase chain reaction (PCR) method results of 96 DNA samples were validated by Sanger sequencing. Primers for PCR were designed at the Oligonucleotide Synthesis Unit of the Genetics department at KFSHRC. Primer details are described in Table 1.

**TABLE 1:** Designed primers used for PCR.

Rs number-product size-annealing temperature	Forward primer	Reverse primer
CYP2C9*3 Rs1057910 404 bp 56°C	TGG​CAG​AAA​CCG​GAG​CCC​CT	GCA​CCT​AAG​AGT​AGC​CAA​ACC​AAT​CTT
*VKORC1* Rs9923231 372 bp 55°C	GGG​AGG​AGC​CAG​CAG​GAG​AGG	AGCGGTGCCATCTCGGC

### 2.5 Data analysis

Data were analyzed using SPSS version 27 (IBM Corp- Chicago- IL- United States). Continuous variables are described as medians, means, and standard deviations. Categorical variables were summarized with numbers and percentages. Hardy-Weinberg equilibrium was assessed using the following equation: p2+2pq + q2 = 1, where p2 = frequency of homozygous wild genotype; 2pq = frequency of heterozygous genotype; q2 = frequency of homozygous mutant genotype.

The chi-squared test was used to compare observed genotype frequencies with expected frequencies. Analysis of variance (ANOVA) was carried out to determine the association between *CYP2C9*, *CYP4F2*, and *VKORC1* genotype and warfarin dose requirement in two groups based on target INR range. Logarithmic transformation was performed to normalize warfarin doses. Data for each target INR group (2.0–3.0 and 2.5–3.5) were analyzed separately. Backward linear regression analysis was performed to identify genetic and clinical factors contributing to warfarin dose requirements. Log-transformed warfarin doses were used as a dependent variable to ensure that the values were normally distributed. Patient age, gender, BSA, smoking status, hypertension (HTN), diabetes mellitus (DM), myocardial infarction (MI), heart failure (HF), hypothyroidism, renal impairment, beta-blockers, antiplatelet, *CYP2C9*, *VKORC1* and *CYP4F2* were treated as independent variables.

## 3 Results

### 3.1 Baseline characteristics

**FIGURE 1:**
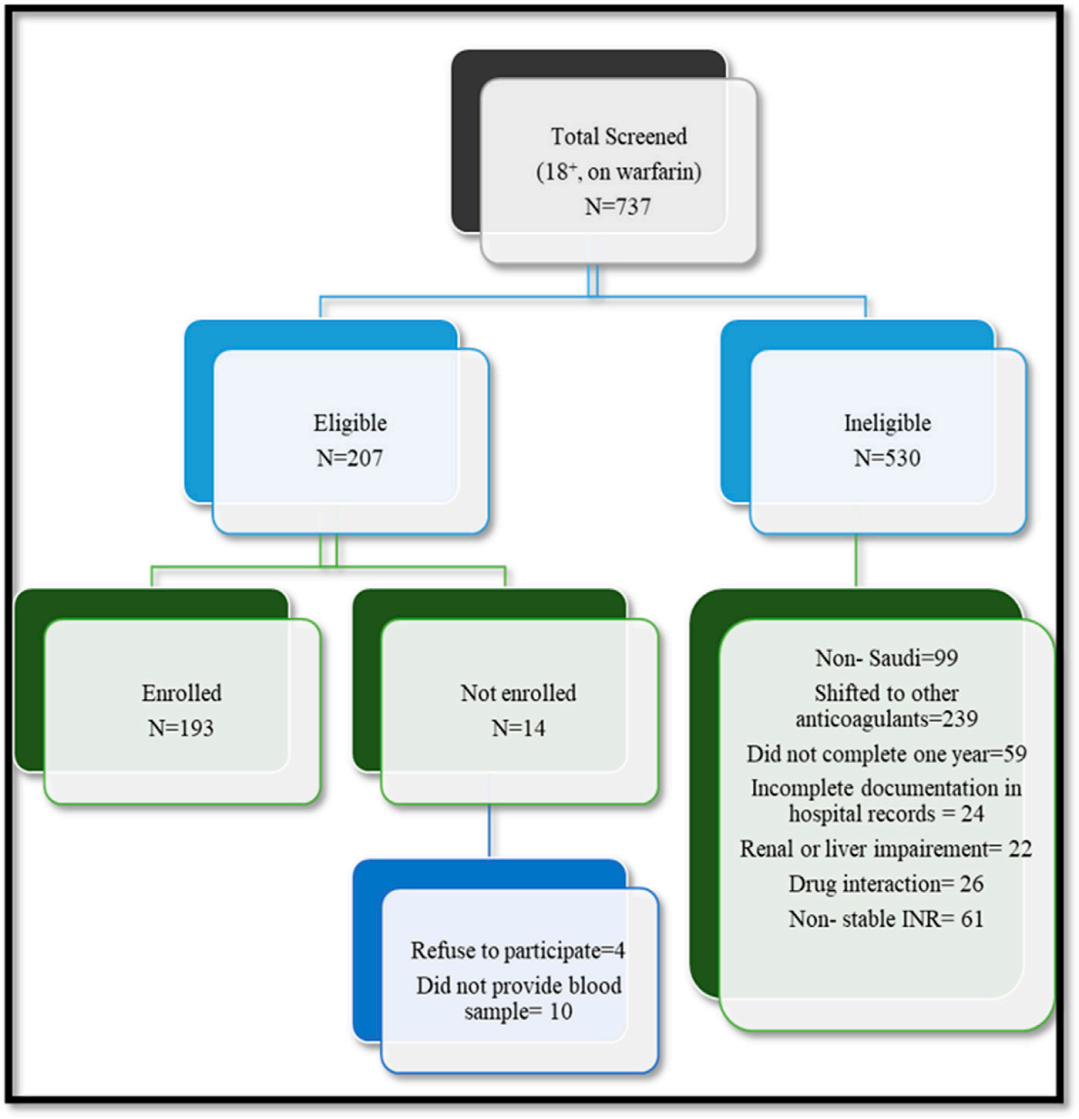
The study flow chart for patients on warfarin enrolled in the study.

Out of 207 patients on long-term warfarin therapy identified for possible study participation, 193 (93.2%) met the inclusion criteria, signed informed consent, and answered the questionnaire, and each provided a blood (5 mL) sample. Figure 1 shows the flow chart for patient recruitment.

Table 2 represents the demographic and clinical characteristics of the patients which were analyzed separately based on the target INR range. Ninety-six patients had mechanical prosthetic heart valves with target INR 2.5–3.5. Their mean (±SD) age was 53 (±13) years (range: 19–92 years; median = 54 years). 52.1% of the participants in this group were female and 40.6% were on warfarin therapy for over 10 years with a mean (±SD, median, range) daily doses of warfarin of 5.86 mg (±3.06, 5, 1–18 mg). The remaining 97 patients in the cohort had a target INR 2.0–3.0. Their mean (±SD) age was 51.6 (±12.75) years (range: 22–88 years; median = 52 years). Approximately 68% of the participants in this group were female. Deep vein thrombosis/pulmonary embolism was the most common cause of warfarin use (45.4%). Other indications for anticoagulation were valvular replacement, atrial fibrillation, stroke and other indications in 24.7%, 17.5%, 7.2 and 5.2 of patients, respectively. About 50% of the participants have received warfarin therapy for more than 10 years. The mean (±SD, median, range) daily doses of warfarin in the group was 6.13 mg (±3.41, 5, 1–18 mg).

**TABLE 2:** Demographic and clinical characteristics of patients who received warfarin therapy.

	2–3 INR group (97 patients)	2.5–3.5 INR group (96 patients)
Characteristics	n (%)	n (%)
Age (years) Mean (±SD, range)	51.6 (±12.75, 22–88)	53 (±13, 19–92)
Gender		
Female Male	66 (68) 31 (32)	50 (52.1) 46 (47.9)
BMI, mean (±SD) BMI = Weight (kg)/height(m)^2^	31.7 (±6.6)	28.8 (±5.47)
Smoking status		
Smoker Nonsmoker	5 (5.2) 92 (94.8)	9 (9.4) 87 (90.6)
Warfarin Indication		
Valve replacement AF DVT or PE Stroke Other	24 (24.7) 17 (17.5) 44 (45.4) 7 (7.2) 5 (5.2)	95 (99) 1 (1)
Duration on warfarin (years)		
1–4 5–10 >10	24 (24.7) 24 (24.7) 49 (50.5)	19 (19.8) 38 (39.6) 39 (40.6)
Daily doses (years) Mean (±SD, median, range)	6.13 mg (±3.41, 5, 1–18 mg)	5.86 mg (±3.06, 5, 1–18 mg)

BMI, Body mass index; AF, Atrial fibrillation; DVT, Deep venous thrombosis; PE, Pulmonary embolism

### 3.2 *CYP2C9*, *VKORC1*, and *CYP4F2* genotype frequencies

Table 3 summarizes the Saudi participants’ genotype distribution for *VKORC1*, CYP2C9*2 and *3 and *CYP4F2*. The *VKORC1*, CYP2C9*2 and *3 and *CYP4F2* genotype were in Hardy-Weinberg equilibrium. The results of PCR were in 100% concordance with those obtained by the Sanger sequencing method.

**TABLE 3:** Genotype frequency in the Saudi general population and study patients.

	Patients on warfarin therapy (n = 193)		
Genotype	n-Frequency (%)	Genotype	n-Frequency (%)
CYP4F2 V433M		CYP2C9	
Homozygous wild CC	67–0.35 (34.7)	*1*1	99–0.51 (51.3)
Heterozygous CT	95–0.49 (49.2)	*1*2	51–0.26 (26.4)
Homozygous mutant TT	31–0.16 (16.1)	*1*3	22–0.12 (11.4)
Wild (C)	0.59 (59.1)	*2*2	11–0.06 (5.7)
Mutant (T)	0.41 (40.9)	*3*3	4–0.02 (2.1)
		*2*3	6–0.03 (3.1)
VKORC1		Wild (*1)	0.7 (70.2)
Homozygous wild GG	49–0.25 (25.4)	Mutant (*2)	0.20 (20.5)
Heterozygous GA	83–0.43 (43)	Mutant (*3)	(9.3)
Homozygous mutant AA	61–0.32 (31.6)		
Wild (G)	0.47 (46.9)		
Mutant (A)	0.53 (53.1)		

*1 is the wild- type allele of CYP2C9, *2 and *3 are variant type alleles.

The results showed that the CYP2C9*1*1 genotype was observed in 51.3% of participants, making it the most prevalent genotype. CYP2C9 *2 (20.5) was more common than CYP2C9*3 (9.3). Approximately 43% of the study patients were heterozygous (GA) for the *VKORC1* genotype, 31% homozygous mutant alleles, and 25% homozygous wild-type genotypes. With regard to *CYP4F2* V433M, 49.2% of the patients were of the heterozygous (CT) genotype, and ∼34% were homozygous wild-type (CC) alleles, while 16% were homozygous mutant (TT) alleles.

### 3.3 Impact of *CYP2C9*, *VKORC1* and *CYP4F2* on warfarin dose requirement

**FIGURE 2:**
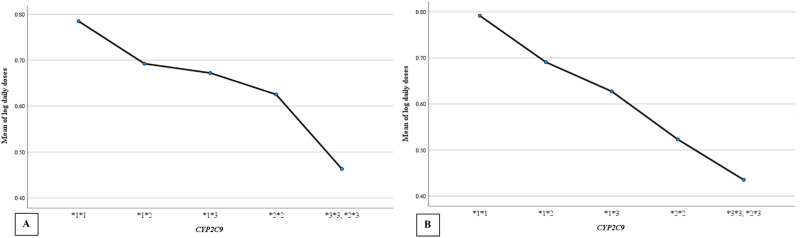
The mean daily warfarin dose requirement between *CYP2C9* wild-type and variant genotypes. **(A)** target INR 2–3, **(B)** target INR 2.5–3.5.

**FIGURE 3:**
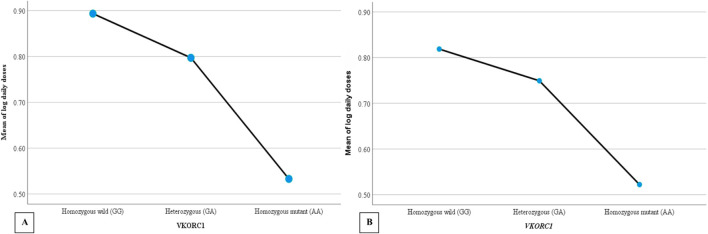
The mean daily warfarin dose requirement between *VKORC1* wild-type and variant genotypes. **(A)** Target INR 2–3, **(B)** Target INR 2.5–3.5.

Warfarin dose requirements were significantly lower in patients with polymorphisms in *CYP2C9* (Figure 2) and *VKORC1* (Figure 3) than in wild-type patients (one way-ANOVA). Additionally, carriers of two mutant alleles had significantly lower dosage requirements than those of one mutant allele. In contrast, polymorphism in *CYP4F2* did not affect warfarin dose requirements (Table 4).

**TABLE 4:** The median daily warfarin doses for polymorphisms in each gene.

		INR target 2–3		INR target 2.5–3.5
Genotype	n	Median warfarin daily dose in mg (IQR)	n	Median warfarin dose in mg (IQR)
CYP2C9 p-value		0.034		<0.001
*1*1	53	5.5 (4.29)	46	6 (2.5)
*1*2	26	5 (3.57)	25	5 (2.90)
*1*3	10	4.5 (3.38)	12	3.75 (3.75)
*2*2	5	3.64 (2.75)	6	3.61 (3.07)
*2*3 and *3*3^a^	3	3.5 (2.3)	7	3 (2)
VKORC1 p-value		(<0.001)		(<0.001)
GG	26	7.25 (6.88)	23	6.5 (3)
GA	37	6 (2.25)	46	5.25 (3)
AA	34	3.5 (1)	27	3.5 (1.29)
CYP4F2 p-value		0.31		0.986
CC	29	5 (2.43)	38	5 (3.09)
CT	51	5.5 (4.5)	44	5 (3.09)
TT	17	5 (3.65)	14	5.2 (2.48)

^a^ Only one, and three patients carried *3*3 in the 2-3 and 2.5–3.5 INR, groups, respectively. Therefore, the data for this genotype were combined with the data for *2 *3 genotype. (INR, International Normalization Ratio).

Backward linear regression analysis was used to identify genetic and clinical factors contributing to warfarin dose requirements using log daily dose as a dependent variable and patients’ age, gender, smoking status, hypertension, diabetes, myocardial infarction, heart failure, hypothyroidism, renal impairment, beta-blockers, antiplatelet, *CYP2C9*, *VKORC1*, and *CYP4F2* as independent variables (Table 5). Age, BSA, *CYP2C9*, and *VKORC1* accounted for 54.6% of the interindividual variability in warfarin dose requirement in patients with a target INR of 2.0–3.0. In patients with a target INR of 2.5–3.5, BSA, *CYP2C9*, and *VKORC1* explained 51.2% of warfarin dose requirements. Age and polymorphisms in *CYP2C9* and *VKORC1* were negatively correlated with warfarin dose, while BSA was positively correlated with dose requirement.

**TABLE 5:** Linear regression analysis for predictors of warfarin dose requirements.

Predictor	B	Standardized coefficient beta	p-value	95% confidence interval
INR 2–3				
CYP2C9	−0.045	−0.226	0.002	−0.073 – −0.017
Age	−0.004	−0.238	0.001	−0.007 – −0.002
VKORC1	−0.174	−0.618	<0.001	−0.214 – −0.135
BSA	0.241	0.225	0.002	0.093–0.388
INR 2.5–3.5				
CYP2C9	−0.066	−0.433	<0.001	−0.088 – −0.043
VKORC1	−0.127	−0.449	<0.001	−0.169 – −0.085
BSA	0.224	0.247	0.001	0.093–0.355

## 4 Discussion

Warfarin use is complicated by the risk of bleeding and thrombosis owing to its narrow therapeutic window and variable dose response. Several demographics, clinical and genetic factors are known to significantly affect response to warfarin therapy. These include mainly patient’s age, gender, body size, comorbidities, concurrent medications, genetics, smoking as well as drug and food interactions (Apostolakis et al., 2013). Clinical factors together with genetic polymorphism in *VKORC1 -*1639G>A, CYP2C9*2, CYP2C9*3 and *CYP4F2* V433M are responsible for 55%–57% of the variability in anticoagulation response to warfarin among Caucasians and 25% in African Americans (Cavallari and Perera, 2012; McDonald et al., 2009; Zhang et al., 2017).

Polymorphisms in the *CYP2C9*, *VKORC1*, and *CYP4F2* genes have been found to contribute to the variability in warfarin dosage requirements in several ethnic groups. It is regarded that patients with polymorphisms in the *VKORC1* gene have reduced expression of the VKOR enzyme. This type of mutation puts patients at higher risk of bleeding associated with VKAs therapy, necessitating lower warfarin doses. CYP2C9 is the major enzyme responsible for warfarin metabolism. Two major *CYP2C9* variant alleles (CYP2C9 *2 and *3) affecting warfarin metabolism have been identified thus far in the literature. Carriers of the CYP2C9*2 and CYP2C9*3 variant alleles require lower warfarin doses than carriers of the wild-type *CYP2C9* allele (Borgiani et al., 2009; Cen et al., 2010; Tatarunas et al., 2014). The CYP2C9*3 variant allele leads to the expression of CYP2C9 with reduced activity to a greater extent than the CYP2C9*2 variant allele. On the other hand, carriers of the *CYP4F2* allele have been reported to require higher warfarin doses (Caldwell et al., 2008). However, *CYP4F2* polymorphism has less impact on warfarin dose than those of the *CYP2C9* and *VKORC1* genes.

People from Saudi Arabia are among the ethnic groups that have not been adequately studied in warfarin pharmacogenetics research. To our knowledge, this is the first study investigating the prevalence of the *CYP4F2 V433M* polymorphism and its impact on the warfarin response among Saudi patients. Moreover, very few studies to date have investigated the prevalence of *CYP2C9* and *VKORC1* and their effect on warfarin dose requirements and anticoagulation response (Alzahrani et al., 2013; Al Ammari et al., 2020; Mirghani et al., 2011; Saour et al., 2011; Mizzi et al., 2016). Therefore, we aimed to investigate the frequency of the VKORC1 –1639G>A, CYP2C9*2 and *3 alleles, and *CYP4F2* (*G1347A*) polymorphism and the association between *VKORC1, CYP2C9* and *CYP4F2* genotypes and warfarin dose requirement in this population.

The frequency of *CYP2C9*, *VKORC1*, and *CYP4F2* genotypes varies among different ethnic populations. Americans and Europeans have higher CY2C9*2 and CY2C9*3 allele frequencies than African-American and Asian populations. The frequency of CYP2C9*1, *2, and * 3 are present in approximately 80%, 13%, and 7% of Caucasian individuals, respectively. Southeast Asians have a somewhat higher percentage of CYP2C9*3 (2%–10%), while CYP2C9*2 is almost absent. CYP2C9*2 (3%–5%) and *3 (1%–2%) polymorphisms are rare in African Americans (Gulseth et al., 2009; Westervelt et al., 2014). CYP2C9*1*1 was the most common genotype, while *3*3 was the least common in our population of Saudi patients. Additionally, the frequencies of the heterozygous alleles *1*2 and *1*3 were higher than those of the homozygous alleles *2*2 and *3*3. CYP2C9 *1*1 was present in 51.3% of patients on warfarin treatment. This was slightly lower than that reported in other studies of Saudi patients of 68.7% (Alzahrani et al., 2013), 64.1% (Mirghani et al., 2011), and 63.4% (Saour et al., 2011). However, the genotypic frequency of 26.4% for CYP2C9 *1*2 in anticoagulated patients was similar to the 26.7% reported by Alzahrani et al. (2013) did not report any patients on warfarin in their study with homozygous mutant allele genotypes (*2*2 and *3*3). The *VKORC1-1639A* variant allele is prevalent in approximately 67%, 40%, and 11% of Asians, African Americans and Caucasians, respectively. In the current study, approximately 43% of participants carried the heterozygous *VKORC1-GA* genotype, 31% carried the homozygous mutant (*AA*) allele, and 25% carried the homozygous wild-type allele (*GG*). This finding is similar to the findings of Al Ammari et al. (2020), who reported the following frequencies: *GA* (45.3%), *AA* (34%), and *GG* (20.7%). Regarding the *CYP4F2 V433M* genotype, 49.2% of the anticoagulated patients carry the heterozygous (*CT*) allele. Almost 34% of the participants carry the homozygous wild-type (*CC*) allele, while 16% carry the homozygous mutant (*TT*) allele. The frequency of the mutant *CYP4F2 T* allele in this study was 40.9%, while it was approximately 30% in white and Asian people and 7% in black individuals (Caldwell et al., 2008).

The association between CYP2C9*2 and *3, *CYP4F2* (*G1347A*), and *VKORC1 –1639G>A* genotypes and warfarin dose requirements varies among populations. Compared to Caucasians, warfarin doses are higher among African Americans and lower among Asians. The main reason is the different gene frequencies reported in different racial groups. For instance, *VKORC1(-1639A*), CYP2C9*2, and *3 alleles have been found to be significantly associated with lower warfarin dose requirements in several populations. The frequency of *VKORC1* (*-1639A*), CYP2C9*2, and *3 is lower in African–Americans than in Asians and Caucasians (Cavallari and Perera, 2012). *CYP4F2* has also been found to affect warfarin dosage requirements in white and Asian population but not in Indians, Egyptians, Brazilians, and Black ethnic groups (Danese et al., 2019). This study found that patients carrying the CYP2C9*2, *3, and *VKORC1 A* allele required lower doses than those carrying the wild-type *CYP2C9* and *VKORC1 G* allele. This study is the first to evaluate the impact of *CYP4F2* polymorphism on warfarin dose requirements and warfarin response in a Saudi patient population. In contrast to *CYP2C9* and *VKORC1*, *CYP4F2* polymorphism was not found to be related to warfarin dose requirement.

There is a lack of research on the impact of the combined effect of clinical and genetic factors on warfarin dosing in the Saudi population. The association between genetic polymorphisms and stable maintenance doses was not properly assessed in previous studies of Saudi patients. Al Ammari et al. (2021) assessed the relationship between polymorphisms in *VKORC1* and warfarin doses during the initiation phase (first 10 days of therapy). Al-Saikhan and colleagues published two separate pieces of research to assess the effects of *CYP2C9* and *VKORC1 1173C>T* SNP on warfarin dose variability in 112 and 164 Saudi patients, respectively. However, the combined effect of genotypes and clinical factors was not addressed (Al-Saikhan and Abd-Elaziz, 2018). Saour et al. (2011) evaluated the effects of *CYP2C9* polymorphism on the requirements of warfarin doses in Saudi individuals who received 2 mg or less of warfarin daily. Associations with doses greater than 2 mg were not assessed.

The present study demonstrated that age, BSA, CYP2C9*2 and *3, and *VKORC1 (–1639G>A*) variants contribute significantly to warfarin dose requirements. Age and variants of *CYP2C9* and *VKORC1* alleles were negatively correlated with warfarin doses, while BSA positively correlated with dosage requirements. It has been reported that *CYP2C9* and *VKORC1* variant alleles cause significant differences in warfarin doses in Caucasians, Asians, and African Americans; however, *VKORC1* is known to be less common in African–Americans (Cavallari and Perera, 2012). The results of this study are similar to those of other previous studies in Caucasian populations. In a study by Kamali et al. (2004), age and CYP2C9*2 and *3 alleles significantly contributed to warfarin dose requirements. Sconce et al. (2005) reported that warfarin doses are negatively correlated with age, body weight, height, body surface area, *VKORC1* (*–1639G>A*), CYP2C9*2 and *3 alleles. Genetic polymorphisms in *CYP2C9* and *VKORC1*, together with age and height, were responsible for 54% of the variation in warfarin dose. Caldwell et al. (2008) suggested that clinical factors and polymorphisms in *CYP2C9* and *VKORC1* explained 54% of the interindividual variability in warfarin response. Bourgeois et al. (2016) performed a prospective cohort study to identify clinical and genetic factors contributing to warfarin response in 711 British patients. The multifactorial analysis of genetic (*VKORC1* and *CYP2C9*) and clinical factors explained 57.89% of the variation in the mean weekly warfarin dose and 56.97% in the stable mean weekly dose. Analyzing 117 studies involving patients of various ethnic origins in a meta-analysis by Jorgensen et al. (2012) revealed that *CYP2C9* and *VKORC1* variant alleles significantly contribute to warfarin dose requirements in most ethnicities. Loebstein et al. (2001) established that older patients on warfarin treatment required significantly lower doses than younger patients. Gage et al. (2008) identified predictors of warfarin dose as *VKORC1 −1639/3673 G>A* (−28%), BSA (+11% per 0.25 m^2^), CYP2C9*3 (−33%), CYP2C9*2 (−19%), age (−7% for each decade), target INR (+11% for every 0.5-unit increase), amiodarone (−22%), smoking status (+10%), race (−9%) and current thrombosis (+7%). Similar to some previous studies (Rieder et al., 2005; Wadelius et al., 2005; Bourgeois et al., 2016), we found that the contribution of *VKORC1* to warfarin dosage requirements was greater than that of *CYP2C9*.

The strengths of this study lie in the rigorous criteria that were used for participant selection. Eligibility criteria included stable INR control based upon a minimum period of 3 months. In addition, patients with factors that might affect anticoagulation control were excluded, all of which are important in determining the appropriate warfarin dose for each patient. Furthermore, sequencing was performed on random samples to validate the results of the real-time PCR with 100% concordance. Both real-time PCR genotyping and sequencing provide accurate genotyping analysis

The main limitation in our study is that sample size calculation was not performed, so the study may lack the power to detect the possible significant impact of *CYP4F2* on warfarin dose requirements. Further studies are required to examine the effect of *CYP4F2* polymorphism on warfarin dose requirements in a larger sample of Saudi patients.

## 5 Conclusion

This work contributes to the existing knowledge of how genetics affects the response to warfarin by providing new data regarding an underrepresented ethnic group, Saudi Arabia. This study showed that Saudi patients with polymorphisms in *CYP2C9* and *VKORC1* required lower warfarin doses than those with the wild-type allele. Integrating patient clinical factors, including age and BSA, and genetic polymorphisms in *CYP2C9* and *VKORC1* provides the best estimation of factors contributing to warfarin dose in this population.

## Data Availability

The raw data supporting the conclusions of this article will be made available by the authors, without undue reservation.

## References

[B1] Al Ammari M. AlBalwi M. Sultana K. Alabdulkareem I. B. Almuzzaini B. Almakhlafi N. S. (2020). The effect of the VKORC1 promoter variant on warfarin responsiveness in the Saudi WArfarin Pharmacogenetic (SWAP) cohort. Sci. Rep. 10 (1), 11613. 10.1038/s41598-020-68519-9 32669629 PMC7363835

[B2] Al Ammari M. AlThiab K. AlJohani M. Sultana K. Maklhafi N. AlOnazi H. (2021). Tele-pharmacy Anticoagulation clinic during COVID-19 pandemic: patient outcomes. Front. Pharmacol. 12p, 652482. 10.3389/fphar.2021.652482 PMC8459665 34566632

[B3] Al-Saikhan F. Abd-Elaziz M. Ashour R. (2018). Influence of vitamin K epoxide reductase complex 1 polymorphism on warfarin therapy in a cohort study of Saudi patients. Int. J. Pharmacol. 18, 414–420. 10.3923/ijp.2018.415.420

[B4] Al-Saikhan F. Abd-Elaziz M. A. R. Hamdy Asho R. Langaee T. (2018). Impact of cytochrome P450 2C9 polymorphism on warfarin therapy in Saudi population. Int. J. Pharmacol. 14, 566–571. 10.3923/ijp.2018.566.571

[B5] Alzahrani A. M. Ragia G. Hanieh H. Manolopoulos V. G. (2013). Genotyping of CYP2C9 and VKORC1 in the Arabic population of Al-ahsa, Saudi Arabia. BioMed Res. Int. 2013, 315980. 10.1155/2013/315980 23586031 PMC3613048

[B6] Apostolakis S. Sullivan R. M. Olshansky B. Lip G. Y. H. (2013). Factors affecting quality of anticoagulation control among patients with atrial fibrillation on warfarin: the SAMe-TT₂R₂ score. Chest 144 (5), 1555–1563. 10.1378/chest.13-0054 23669885

[B7] Borgiani P. Ciccacci C. Forte V. Sirianni E. Novelli L. Bramanti P. (2009). CYP4F2 genetic variant (rs2108622) significantly contributes to warfarin dosing variability in the Italian population. Pharmacogenomics 10 (2), 261–266. 10.2217/14622416.10.2.261 19207028

[B8] Bourgeois S. Jorgensen A. Zhang E. J. Hanson A. Gillman M. S. Bumpstead S. (2016). A multi-factorial analysis of response to warfarin in a UK prospective cohort. Genome Med. 8 (1), 2. 10.1186/s13073-015-0255-y 26739746 PMC4702374

[B9] Caldwell M. D. Awad T. Johnson J. A. Gage B. F. Falkowski M. Gardina P. (2008). CYP4F2 genetic variant alters required warfarin dose. Blood 111 (8), 4106–4112. 10.1182/blood-2007-11-122010 18250228 PMC2288721

[B10] Cavallari L. H. Perera M. A. (2012). The future of warfarin pharmacogenetics in under-represented minority groups. Future Cardiol. 8 (4), 563–576. 10.2217/fca.12.31 22871196 PMC3463230

[B11] Cen H.-J. Zeng W.-T. Leng X.-Y. Huang M. Chen X. Li J.-L. (2010). CYP4F2 rs2108622: a minor significant genetic factor of warfarin dose in Han Chinese patients with mechanical heart valve replacement. Br. J. Clin. Pharmacol. 70 (2), 234–240. 10.1111/j.1365-2125.2010.03698.x 20653676 PMC2911553

[B12] Danese E. Raimondi S. Montagnana M. Tagetti A. Langaee T. Borgiani P. (2019). Effect of CYP4F2, VKORC1, and CYP2C9 in influencing coumarin dose: a single-patient data meta-analysis in more than 15,000 individuals. Clin. Pharmacol. Ther. 105 (6), 1477–1491. 10.1002/cpt.1323 30506689 PMC6542461

[B13] Gage B. F. Eby C. Johnson J. A. Deych E. Rieder M. J. Ridker P. M. (2008). Use of pharmacogenetic and clinical factors to predict the therapeutic dose of warfarin. Clin. Pharmacol. Ther. 84 (3), 326–331. 10.1038/clpt.2008.10 18305455 PMC2683977

[B14] Gulseth M. P. Grice G. R. Dager W. E. (2009). Pharmacogenomics of warfarin: uncovering a piece of the warfarin mystery. Am. J. health-system Pharm. 66 (2), 123–133. 10.2146/ajhp080127 19139476

[B15] Homme M. B. Reynolds K. K. Valdes R. Linder M. W. (2008). Dynamic pharmacogenetic models in anticoagulation therapy. Clin. Laboratory Med. 28 (4), 539–552. 10.1016/j.cll.2008.10.002 19059061

[B16] Jorgensen A. L. FitzGerald R. J. Oyee J. Pirmohamed M. Williamson P. R. (2012). Influence of CYP2C9 and VKORC1 on patient response to warfarin: a systematic review and meta-analysis. PloS one 7 (8), e44064. 10.1371/journal.pone.0044064 22952875 PMC3430615

[B32] Kalman L. V. Agundez J. Appell M. L. Black J. L. Bell G. C. Boukouvala S. (2016). Pharmacogenetic allele nomenclature: International workgroup recommendations for test result reporting. Clin Pharmacol Ther, 99, 172–185. 10.1002/cpt.280 26479518 PMC4724253

[B17] Kamali F. Khan T. I. King B. P. Frearson R. Kesteven P. Wood P. (2004). Contribution of age, body size, and CYP2C9 genotype to anticoagulant response to warfarin. Clin. Pharmacol. Ther. 75 (3), 204–212. 10.1016/j.clpt.2003.10.001 15001972

[B18] Kamali F. Wynne H. (2010). Pharmacogenetics of warfarin. Annu. Rev. Med. 61pp, 63–75. 10.1146/annurev.med.070808.170037 19686083

[B33] Lane S. Al-Zubiedi S. Hatch E. Matthews I. Jorgensen A. L. Deloukas P. (2012). The population pharmacokinetics of R- and S- warfarin: effect of genetic and clinical factors. Br J Clin Pharmacol, 73, 66–76. 10.1111/j.1365-2125.2011.04051.x 21692828 PMC3248257

[B19] Loebstein R. Yonath H. Peleg D. Almog S. Rotenberg M. Lubetsky A. (2001). Interindividual variability in sensitivity to warfarin--Nature or nurture? Clin. Pharmacol. Ther. 70 (2), 159–164. 10.1067/mcp.2001.117444 11503010

[B20] McDonald M. G. Rieder M. J. Nakano M. Hsia C. K. Rettie A. E. (2009). CYP4F2 is a vitamin K1 oxidase: an explanation for altered warfarin dose in carriers of the V433M variant. Mol. Pharmacol. 75 (6), 1337–1346. 10.1124/mol.109.054833 19297519 PMC2684883

[B21] Mirghani R. A. Chowdhary G. Elghazali G. (2011). Distribution of the major cytochrome P450 (CYP) 2C9 genetic variants in a Saudi population. Basic and Clin. Pharmacol. and Toxicol. 109 (2), 111–114. 10.1111/j.1742-7843.2011.00692.x 21371265

[B22] Mizzi C. Dalabira E. Kumuthini J. Dzimiri N. Balogh I. Başak N. (2016). A European spectrum of pharmacogenomic biomarkers: implications for clinical pharmacogenomics. PloS one 11 (9), e0162866. 10.1371/journal.pone.0162866 27636550 PMC5026342

[B34] Moyer T. P. P. O'Kane D. J. P. Baudhuin L. M. P. Wiley C. L. P. Fortini A. M. D. Fisher P. K. M. A. (2009). Warfarin Sensitivity Genotyping: A Review of the Literature and Summary of Patient Experience. Mayo Clin Proc, 84, 1079–1094. 10.4065/mcp.2009.0278 19955245 PMC2787394

[B23] Niinuma Y. Saito T. Takahashi M. Tsukada C. Ito M. Hirasawa N. (2014). Functional characterization of 32 CYP2C9 allelic variants. Pharmacogenomics J. 14 (2), 107–114. 10.1038/tpj.2013.22 23752738

[B35] Rieder M. J. Reiner A. P. Gage B. F. Nickerson D. A. Eby C. S. Mcleod H. L. (2005). Effect of VKORC1 haplotypes on transcriptional regulation and warfarin dose. N Engl J Med, 352, 2285–2293. 10.1056/NEJMoa044503 15930419

[B24] Saour J. N. Shereen A. W. Saour B. J. Mammo L. A. (2011). CYP2C9 polymorphism studies in the Saudi population. Saudi Med. J. 32 (4), 347–352. 21483991

[B25] Sconce E. A. Khan T. I. Wynne H. A. Avery P. Monkhouse L. King B. P. (2005). The impact of CYP2C9 and VKORC1 genetic polymorphism and patient characteristics upon warfarin dose requirements: proposal for a new dosing regimen. Blood 106 (7), 2329–2333. 10.1182/blood-2005-03-1108 15947090

[B26] Scott S. A. Khasawneh R. Peter I. Kornreich R. Desnick R. J. (2010). Combined CYP2C9, VKORC1 and CYP4F2 frequencies among racial and ethnic groups. Pharmacogenomics 11 (6), 781–791. 10.2217/pgs.10.49 20504253 PMC2904527

[B27] Tatarunas V. Lesauskaite V. Veikutiene A. Grybauskas P. Jakuska P. Jankauskiene L. (2014). The effect of CYP2C9, VKORC1 and CYP4F2 polymorphism and of clinical factors on warfarin dosage during initiation and long-term treatment after heart valve surgery. J. thrombosis thrombolysis 37 (2), 177–185. 10.1007/s11239-013-0940-x 23677510

[B36] Wadelius M. Chen L. Y. Downes K. Ghori J. Hunt S. Eriksson N. (2005). Common VKORC1 and GGCX polymorphisms associated with warfarin dose. Pharmacogenomics J. 5, 262–270. 10.1038/sj.tpj.6500313 15883587

[B28] Wen M.-S. Lee M. T. M. (2013). Warfarin pharmacogenetics: new life for an old drug. Acta Cardiol. Sin. 29 (3), 235–242. 27122712 PMC4804835

[B29] Westervelt P. Cho K. Bright D. R. Kisor D. F. (2014). Drug-gene interactions: inherent variability in drug maintenance dose requirements. P and T a peer-reviewed J. formulary Manag. 39 (9), 630–637. PMC4159057 25210416

[B30] Wigle P. Hein B. Bloomfield H. E. Tubb M. Doherty M. (2013). Updated guidelines on outpatient anticoagulation. Am. Fam. physician 87 (8), 556–566. 23668445

[B31] Zhang J. E. Klein K. Jorgensen A. L. Francis B. Alfirevic A. Bourgeois S. (2017). Effect of genetic variability in the CYP4F2, CYP4F11, and CYP4F12 genes on liver mRNA levels and warfarin response. Front. Pharmacol. 8p, 323. 10.3389/fphar.2017.00323 PMC5449482 28620303

